# Supplementary material for: Heat Shock Factor 1 Directly Regulates Postsynaptic Scaffolding PSD-95 in Aging and Huntington’s Disease and Influences Striatal Synaptic Density
Source: Int J Mol Sci. 2021 Dec 4;22(23):13113. doi: 10.3390/ijms222313113 (PMC8657899; doi:10.3390/ijms222313113)
Supplement: Supplementary file 1 [file ijms-22-13113-s001.zip › ijms-1463827-supplementary.pdf]

International Journal of *Molecular Sciences*

MDPI

Article

Heat Shock Factor 1 Directly Regulates Postsynaptic Scaffolding PSD-95 in Aging and Huntington's Disease and Influences Striatal Synaptic Density

Nicole Zarate, Taylor A. Intihar, Dahyun Yu, Jacob Sawyer, Wei Tsai, Maha Syed, Luke Carlson and Rocio Gomez-Pastor *

Supplementary Materials

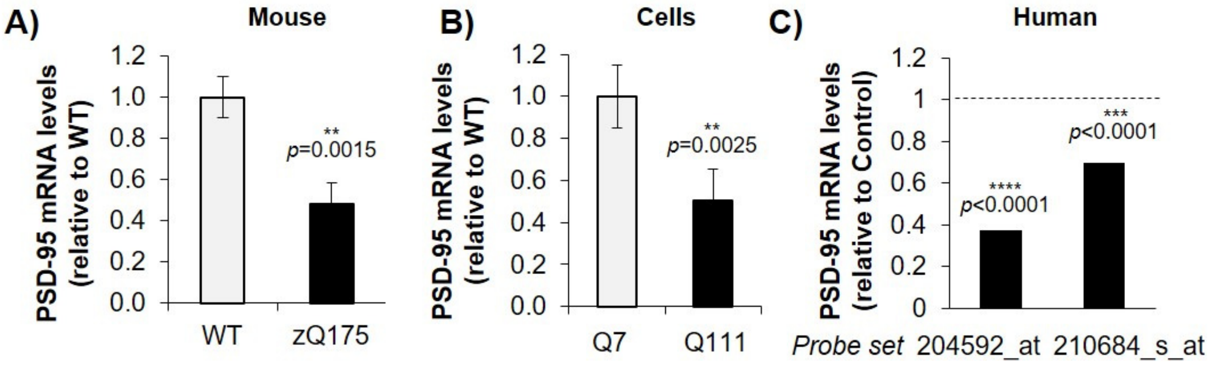

Figure S1. PSD-95 mRNA levels are decreased in HD. PSD-95 mRNA levels in (**A**) the striatum of WT (*n* = 5) and zQ175 (*n* = 6) mice at 6 months, (**B**) the immortalized striatal murine cell lines ST*Hdh*Q7 and ST*Hdh*Q111 and (**C**) in the caudate of patients with HD (*n* = 44) relative to unaffected individuals (*n* = 36) obtained from Hodges et al., [6]. Data is shown as Log2 FC from two different probe sets. Error bars denote mean ± SEM. Unpaired Student's t-test in A and B. * $p < 0.05$, ** $p < 0.01$, *** $p < 0.001$, **** $p < 0.0001$.

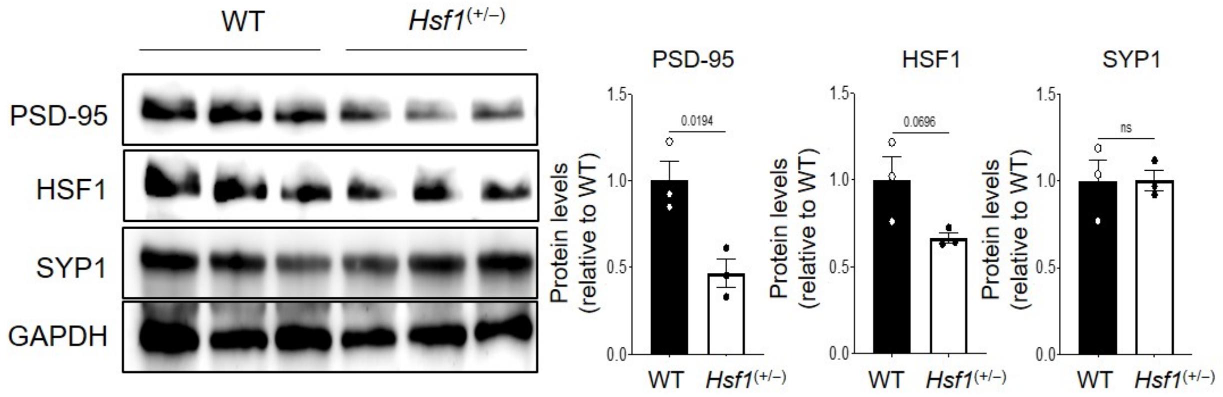

Figure S2. PSD-95 protein levels are decreased in cortices of Hsf1(+/−) mice. (**A**) Immunoblotting in the cortex of WT Hsf1 (+/−) mice (*n* = 3) mice at 6 months. GAPDH is used as a loading control. (**B**) Protein levels measured using Image J analyses from images in A. Data is normalized to GAPDH levels and relativized to WT. Error bars denote mean ± SEM. Unpaired Student's *t*-test. * $p < 0.05$.

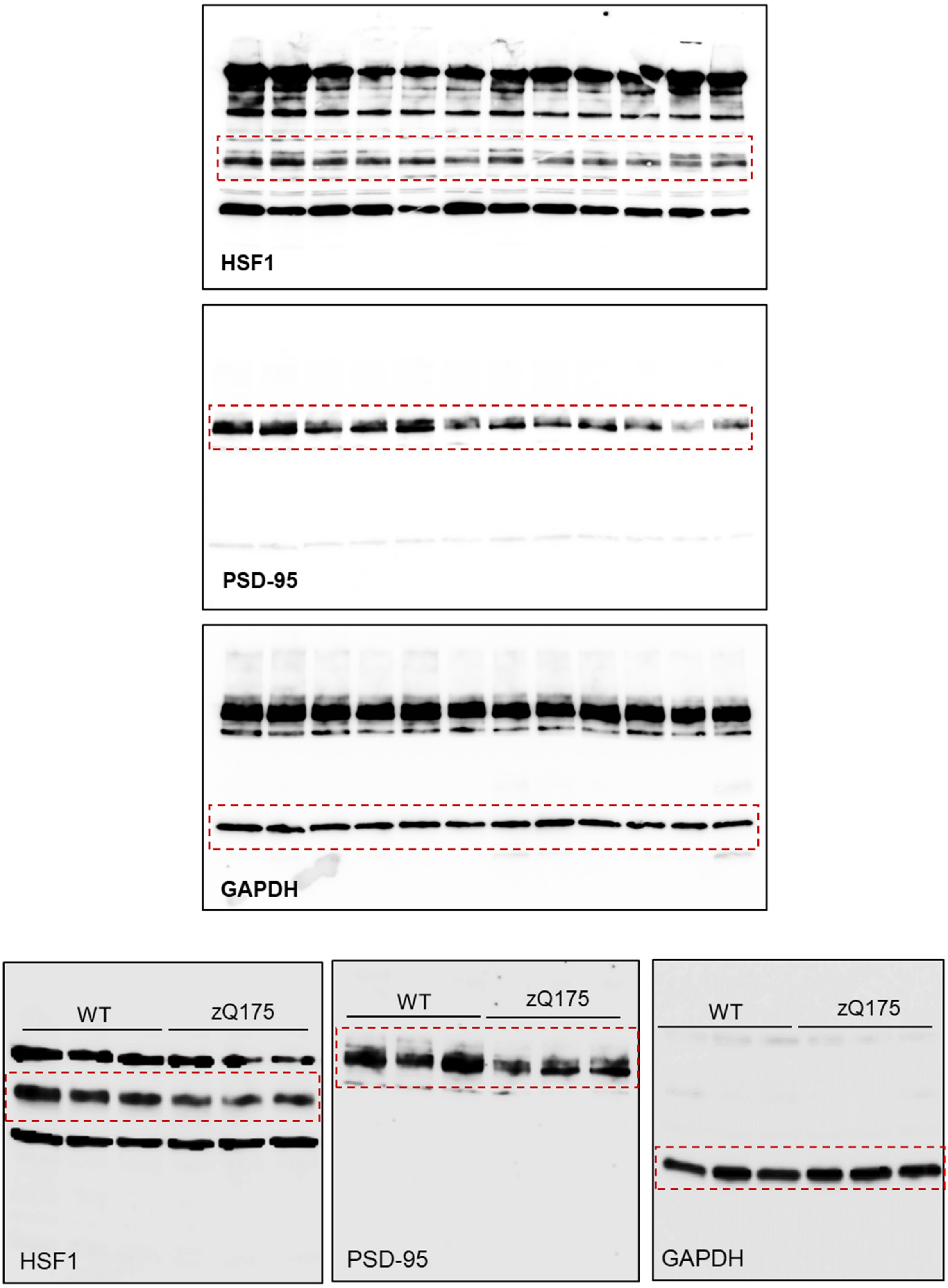

Figure S3. Uncropped blots of Figure 1A,E.

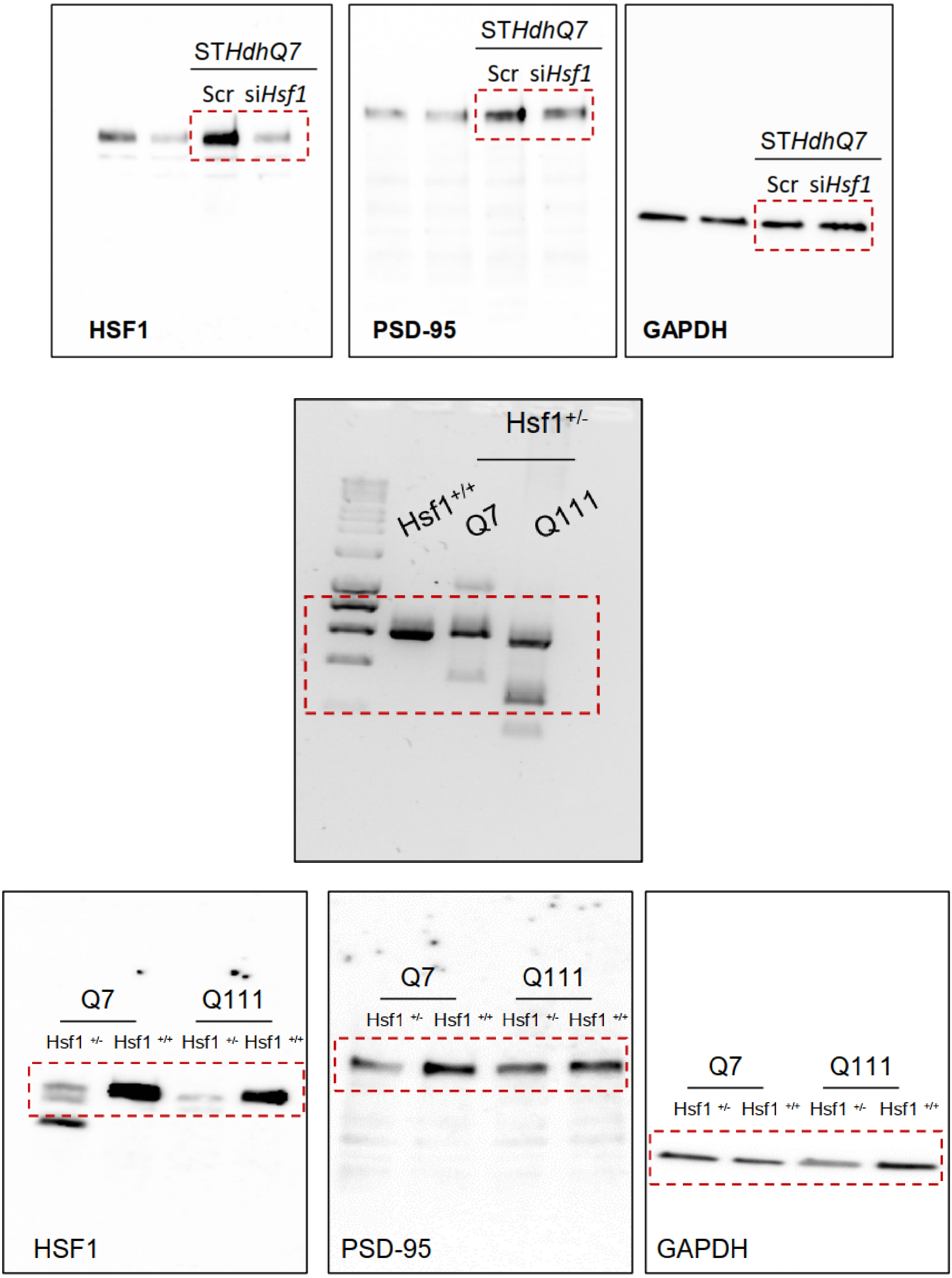

Figure S4. Uncropped blots of Figure 2A,D,E.

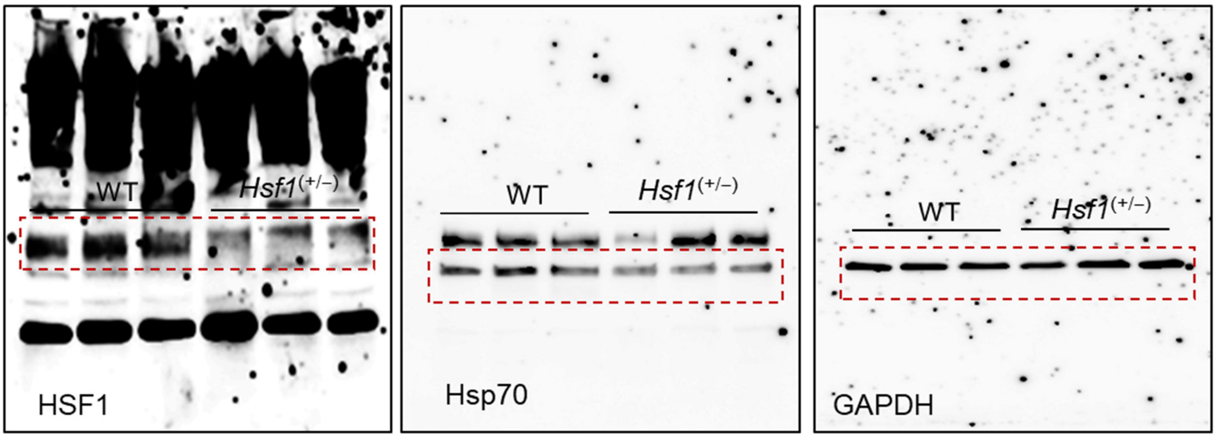

Figure S5. Uncropped blots of Figure 3A.
